# Flt3 agonist enhances immunogenicity of arenavirus vector-based simian immunodeficiency virus vaccine in macaques

**DOI:** 10.1128/jvi.00294-24

**Published:** 2024-06-03

**Authors:** Archana Vidya Boopathy, Anurag Nekkalapudi, Janette Sung, Sophie Schulha, Debi Jin, Bhawna Sharma, Sarah Ng, Sabrina Lu, Raphaela Wimmer, Silpa Suthram, Sarah Ahmadi-Erber, Henning Lauterbach, Klaus K. Orlinger, Magdeleine Hung, Brian Carr, Christian Callebaut, Romas Geleziunas, Michelle Kuhne, Sarah Schmidt, Brie Falkard

**Affiliations:** 1 Clinical Virology, Gilead Sciences, Inc., Foster, California, USA; 2 Drug Metabolism, Gilead Sciences, Inc., Foster, California, USA; 3 Virology, Hookipa Pharma Inc., New York, New York, USA; 4 Protein Therapeutics, Gilead Sciences, Inc., Foster, California, USA; 5 Discovery Virology, Gilead Sciences, Inc., Foster, California, USA; 6 Oncology, Gilead Sciences, Inc., Foster, California, USA; 7 Bioinformatics, Gilead Sciences, Inc., Foster, California, USA; 8 Global Research and Development, Hookipa Pharma Inc., New York, New York, USA; Ulm University Medical Center, Ulm, Germany

**Keywords:** HIV/SIV vaccine, Flt3 agonism, arenavirus vectors, immunogenicity

## Abstract

Arenaviral vaccine vectors encoding simian immunodeficiency virus (SIV) immunogens are capable of inducing efficacious humoral and cellular immune responses in nonhuman primates. Several studies have evaluated the use of immune modulators to further enhance vaccine-induced T-cell responses. The hematopoietic growth factor Flt3L drives the expansion of various bone marrow progenitor populations, and administration of Flt3L was shown to promote expansion of dendritic cell populations in spleen and blood, which are targets of arenaviral vectors. Therefore, we evaluated the potential of Flt3 signaling to enhance the immunogenicity of arenaviral vaccines encoding SIV immunogens (SIV_SME543_ Gag, Env, and Pol) in rhesus macaques, with a rhesus-specific engineered Flt3L-Fc fusion protein. In healthy animals, administration of Flt3L-Fc led to a 10- to 100-fold increase in type 1 dendritic cells 7 days after dosing, with no antidrug antibody (ADA) generation after repeated dosing. We observed that administration of Flt3L-Fc fusion protein 7 days before arenaviral vaccine increased the frequency and activation of innate immune cells and enhanced T-cell activation with no treatment-related adverse events. Flt3L-Fc administration induced early innate immune activation, leading to a significant enhancement in magnitude, breadth, and polyfunctionality of vaccine-induced T-cell responses. The Flt3L-Fc enhancement in vaccine immunogenicity was comparable to a combination with αCTLA-4 and supports the use of safe and effective variants of Flt3L to augment therapeutic vaccine-induced T-cell responses.

**IMPORTANCE:**

Induction of a robust human immunodeficiency virus (HIV)-specific CD4^+^ and CD8^+^ T-cell response through therapeutic vaccination is considered essential for HIV cure. Arenaviral vaccine vectors encoding simian immunodeficiency virus (SIV) immunogens have demonstrated strong immunogenicity and efficacy in nonhuman primates. Here, we demonstrate that the immunogenicity of arenaviral vectors encoding SIV immunogens can be enhanced by administration of Flt3L-Fc fusion protein 7 days before vaccination. Flt3L-Fc-mediated increase in dendritic cells led to robust improvements in vaccine-induced T- and B-cell responses compared with vaccine alone, and Flt3L-Fc dosing was not associated with any treatment-related adverse events. Importantly, immune modulation by either Flt3L-Fc or αCTLA-4 led to comparable enhancement in vaccine response. These results indicate that the addition of Flt3L-Fc fusion protein before vaccine administration can significantly enhance vaccine immunogenicity. Thus, safe and effective Flt3L variants could be utilized as part of a combination therapy for HIV cure.

## INTRODUCTION

Human immunodeficiency virus (HIV) affects millions of people globally and is one of the leading causes of morbidity and mortality (1, 2). In the United States, there are 1.1 million individuals infected with HIV and close to 30,000 new infections annually (3). Although antiretroviral therapy (ART) has been highly successful in treating HIV, pre-exposure prophylaxis, and preventing disease progression, ART medications require lifelong adherence, and a functional cure for HIV remains an unmet medical need. Several studies in nonhuman primates (NHPs) have demonstrated the critical role of CD4^+^ and CD8^+^ T cells in HIV control (4–6). The durable HIV control observed in elite controllers and post-treatment controllers is associated with robust HIV-specific CD8^+^ T-cell responses (7). A therapeutic vaccine capable of stimulating such cellular immune responses could be considered critical for HIV cure. Yet, the latent HIV reservoir and high viral diversity, as well as the limited functionality and durability of vaccine-induced T-cell responses, are key challenges in development of a therapeutic HIV vaccine.

Engineered viral vectors based on adenoviruses, poxviruses, arenaviruses, and alphaviruses are currently explored in vaccine delivery to stimulate antigen-specific T-cell responses against cancer and infectious diseases, including HIV (8, 9). However, early clinical trials utilizing viral vector-based vaccines to induce CD8^+^ T-cell responses demonstrated limited success in HIV control (10–12). Arenaviruses including Pichinde virus (PICV) and lymphocytic choriomeningitis virus (LCMV) are bi-segmented RNA viruses. Given the low seroprevalence of neutralizing antibodies to LCMV and rare, asymptomatic PICV infection in humans, limited prior exposure to these arenaviruses makes them good candidates for vector-based vaccines. Preclinical studies have shown that these arenaviral vectors can preferentially infect and activate dendritic cells (DCs), monocytes, and macrophages over T and B cells, leading to antigen-specific T-cell responses (13, 14). Early clinical trials utilizing PICV- and LCMV-based vectors have shown robust induction of antigen-specific T-cell responses and antitumor activity (15, 16). We have recently shown that these arenaviral vectors are immunogenic and efficacious in a simian immunodeficiency virus (SIV) model of infection in rhesus macaques with a schedule of an initial prime followed by a 12-week boost, with two subsequent boosts every 8 weeks (13, 17). However, the potential of immune modulation in augmenting the immunogenicity of arenaviral vectors has not been investigated in this model.

Dendritic cells (DCs) play a key role in orchestrating innate and adaptive immune responses after infection or vaccination. DC-mediated antigen uptake and presentation to CD8^+^ and CD4^+^ T cells is critical for induction of robust antigen-specific T- and B-cell responses. To improve immunogenicity of protein and subunit vaccines, DC targeting via conjugation to anti-DEC205 monoclonal antibodies (mAb) or CD40L for CD40-mediated DC maturation has been explored. HIV p24 conjugated to anti-DEC205 mAb showed enhanced immunogenicity in preclinical studies, and HIV envelope gp140 trimer fused to CD40L activated DCs and enhanced T-cell priming in *in vitro* studies (18, 19). Unlike single antigen-specific responses, therapeutic vaccines aim to generate a broad immune response to several conserved immunogens. Therefore, the use of immunomodulatory approaches that can expand DCs has been evaluated as an alternative approach to augment vaccine efficacy.

More recently, Flt3L-Flt3 signaling to expand DCs was studied in several preclinical and clinical studies in immuno-oncology (20). Flt3L is a hematopoietic growth factor that binds to Flt3 expressed on myeloid and lymphoid progenitor cells in the bone marrow and terminally differentiated DCs. Flt3L-Flt3 signaling is required for differentiation, expansion, and maintenance of DCs in peripheral and lymphoid organs (21). Clinical trials in oncology have identified a correlation between intra-tumoral type 1 DCs and level of T-cell infiltration. Therefore, strategies to increase type 1 conventional dendritic cell (cDC1) frequency are being evaluated in the clinic, including the soluble Flt3 ligand CDX-301, dosed for 5–14 consecutive days (22). The use of extended half-life Flt3 agonists as a single agent to improve vaccine efficacy has not been investigated. In this study, we evaluated whether Flt3L can increase arenaviral vaccine immunogenicity based on the ability of arenaviral vectors to preferentially infect DCs and the potential of Flt3 agonism to increase DC expansion and maintenance.

## RESULTS

### PK/PD of Flt3L-Fc fusion protein in healthy rhesus macaques

**Fig 1:**
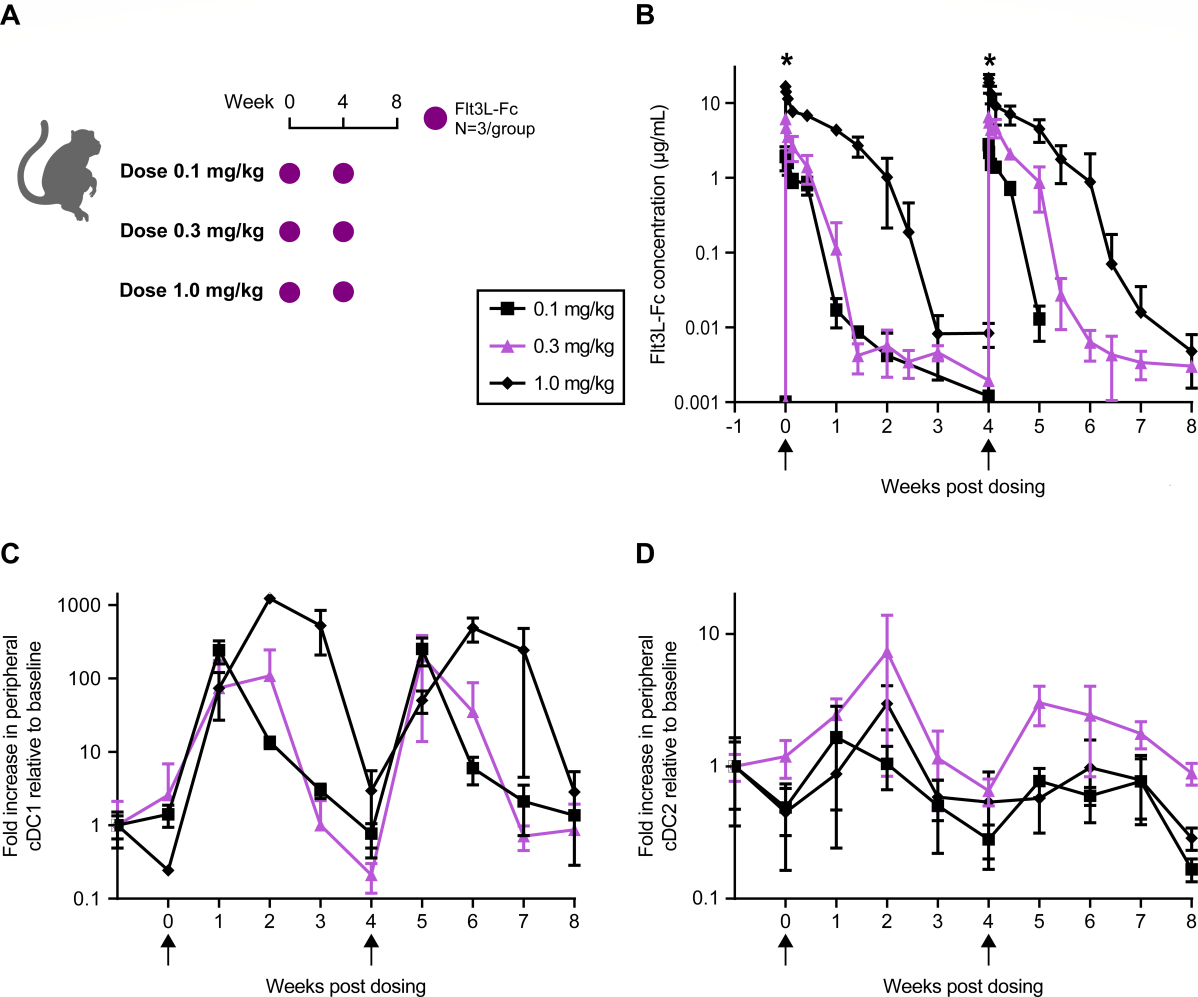
PK/PD of Flt3L-Fc in naïve rhesus. (**A**) Schematic of study design in NHPs. (**B**) Serum concentration of Flt3L-Fc dosed at 0.1, 0.3, and 1.0 mg/kg on day 0 (week 0) and day 28 (week 4). Peripheral dendritic cell subsets: (**C**) cDC1s (CD3^−^CD20^−^CD56^−^HLADR^+^CD123^−^CD11c^hi^Clec9A^+^CD1c^−^) and (**D**) cDC2s (CD3^−^CD20^−^CD56^−^HLADR^+^CD123^−^CD11c^hi^Clec9A^−^CD1c^+^) in peripheral blood measured by whole blood flow cytometry during the study relative to baseline. *N* = 3/group. Data are mean ± standard deviation (SD). *P < 0.05. Statistical analysis by Kruskal-Wallis test with Dunn’s multiple comparisons test at peak time points in B–D.

Flt3L-Fc fusion protein is an engineered recombinant protein designed to bind to Flt3 in rhesus macaques. The cellular potency of Flt3L-Fc was evaluated using a Flt3-expressing cell line (AML5), which is dependent on Flt3 agonism for proliferation. Flt3L-Fc induced dose-dependent proliferation of AML5 cells with an average half-maximal effective concentration (EC_50_) of 0.027 nM in three independent experiments (Fig. S1A and B). To determine the dose of Flt3L-Fc to be used in combination with the artPICV/artLCMV vaccine, a dose-finding pharmacokinetic/pharmacodynamic (PK/PD) study was performed in healthy rhesus macaques. NHPs were administered 0.1, 0.3, or 1.0 mg/kg of Flt3L-Fc on days 0 and 28 of study, as shown in Fig. 1A. Serum concentration of Flt3L-Fc shows maximum concentration (C_max_) at 30 minutes after dosing in all three groups, with significantly higher C_max_ in animals administered 1.0 mg/kg compared with the other two groups (*P* < 0.05, Fig. 1B). Flt3L-Fc serum concentrations returned to baseline by day 28 with significant target-mediated drug disposition response. Importantly, no antidrug antibody (ADA) responses were observed in any of the animals after the two doses of Flt3L-Fc (Fig. S2). Studies have shown that Flt3L-Flt3 interaction leads to increases in cDC1s in peripheral blood (20). We observed a robust increase in cDC1s from baseline at 7 days after administration of 0.1, 0.3, or 1.0 mg/kg of Flt3L-Fc (median fold increase 86-fold at week 1 and 68-fold at week 5; Fig. 1C) with no difference in magnitude of fold increase between the three doses tested. This increase in cDC1s shows a trend toward dose-dependent durability, with a significantly elevated response maintained until day 14 and a return to baseline by day 28, observed after each of the two doses. A similar trend in increase of type 2 dendritic cells (cDC2s) is observed (Fig. 1D). In summary, Flt3L-Fc expanded peripheral cDC1s in rhesus at all doses tested. Flt3L-Fc dose of 0.3 mg/kg resulted in a 33-fold increase in cDC1s at 7 days after the first dose and a 231-fold increase at 7 days after the second dose as compared with baseline (week minus one), along with a trend toward greater induction of cDC2s compared with the other doses tested. Therefore, a dose of 0.3 mg/kg was chosen for subsequent testing in combination with an arenaviral vector vaccine encoding SIV immunogens in NHPs.

### PK/PD of Flt3L-Fc in combination with arenaviral vaccine vectors expressing SIV immunogens

**Fig 2:**
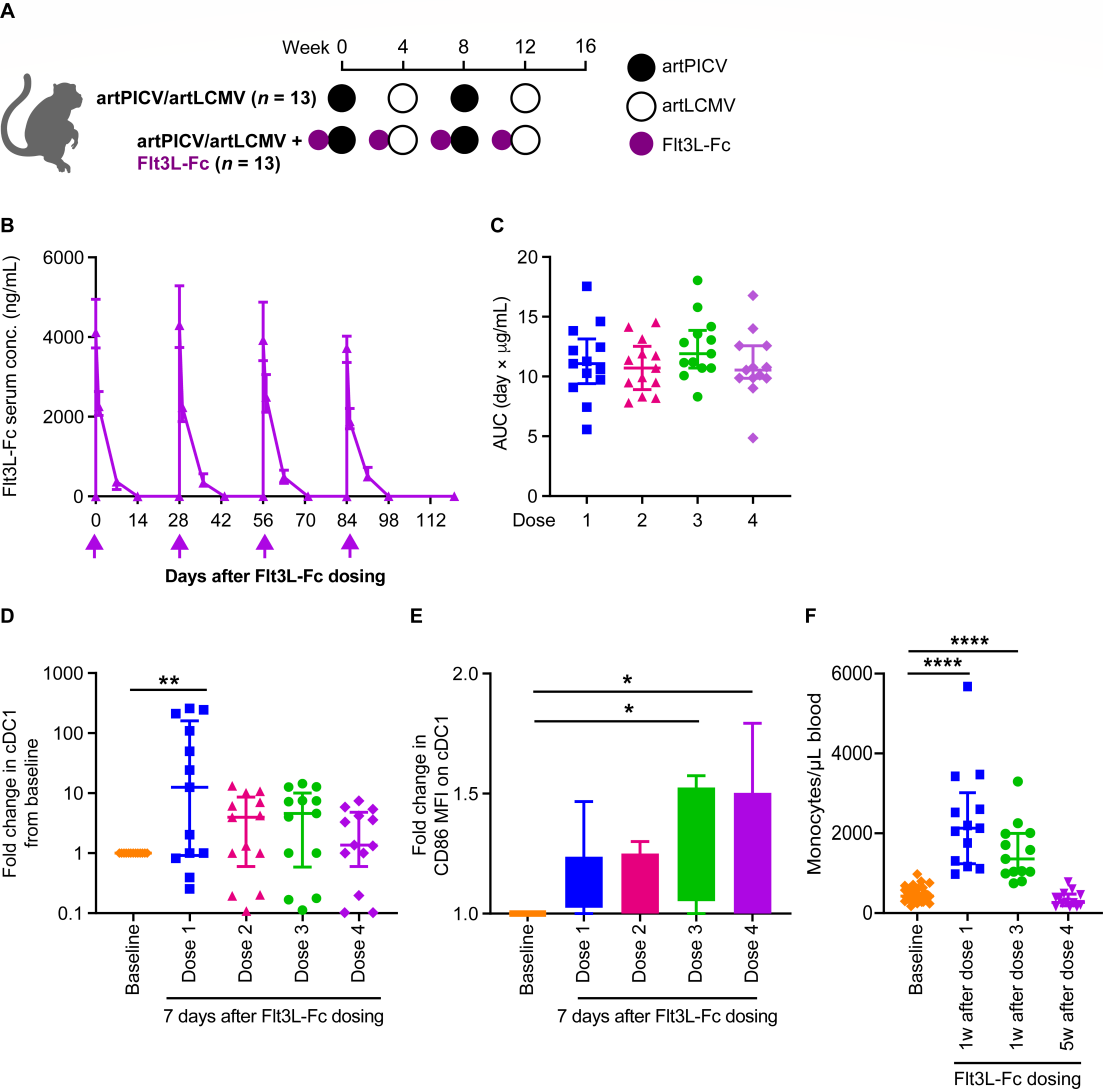
PK/PD of Flt3L-Fc in NHPs administered Flt3L-Fc in combination with the artPICV/artLCMV vaccine. (**A**) Schematic of study design. (**B**) Serum concentration of Flt3L-Fc in rhesus during study. (**C**) AUC for Flt3L-Fc exposure after each dose. (**D**) Fold change in cDC1s in peripheral blood measured by whole blood flow cytometry 7 days after Flt3L-Fc dosing during the study relative to baseline. (**E**) Fold change in median fluorescence intensity (MFI) of activation marker CD86 on cDC1. (**F**) Monocytes in peripheral blood were quantified by complete blood count analysis. *N* = 13/group. Data are median ± interquartile range (IQR). *P < 0.05, **P < 0.01, ****P < 0.0001. Friedman’s test (one-way ANOVA with repeated measures) with Dunn’s post-test for multiple comparisons in D and E, and Kruskal–Wallis test with Dunn’s post-test for multiple comparisons in F.

Arenaviral vaccine vectors have been shown to induce robust vaccine-specific CD4^+^ and CD8^+^ T-cell responses in NHPs (23, 24). Viral vectors evaluated in this study (artPICV based on Pichinde virus strain p18 and artLCMV based on lymphocytic choriomeningitis virus strain cl13) are engineered to express SIV_SME543_ Gag, Env, and Pol immunogens, as previously described (15, 17). To closely examine the ability of Flt3L-Fc to augment the immunogenicity of arenaviral SIV vaccines, naïve Indian-origin rhesus monkeys (*Macaca mulatta*) were immunized in a suboptimal dosing regimen of every 4 weeks by intramuscular injection of the artPICV and artLCMV vectors. Longer dosing intervals between artPICV and artLCMV vectors (8–12 weeks) have been shown to result in robust vaccine immunogenicity in NHPs (17). Animals were immunized with vectors expressing SIV_SME543_ Gag, Env, and Pol immunogens alone, or vaccinated 7 days after Flt3L-Fc intravenous administration (*n* = 13/group; Fig. 2A). No difference in body weight was observed between the two groups (Fig. S3). The serum PK profile was evaluated after administration of each of the four doses of Flt3L-Fc and showed no significant difference in serum concentration after each dose (Fig. 2B and C). The lack of a significant decrease in C_max_ or area under the curve (AUC) after each of the four doses of Flt3L-Fc suggests that ADA responses to Flt3L-Fc are not being induced with repeated dosing. We observed a significant increase in cDC1s in peripheral blood 7 days after the first dose of Flt3L-Fc; however, repeated dosing resulted in a smaller increase in magnitude of circulating cDC1s (Fig. 2D). Interestingly, we observed a significant increase in expression of DC activation marker CD86 after repeated Flt3L-Fc dosing, indicating robust expansion and activation of DCs by Flt3L-Fc (Fig. 2E). An increase in circulating monocytes has been observed as a PD marker after short half-life Flt3L dosing in early clinical trials (22). Here, we observed a similar increase in circulating monocytes 1 week after Flt3L-Fc dosing, with monocyte counts returning to baseline 5 weeks after the last Flt3L-Fc dose (Fig. 2F). In parallel, volumetric cell counts were analyzed at weeks 0, 8, and 16 (Table S1). These results demonstrate the ability of Flt3L-Fc in transiently expanding circulating cDC1s, in combination with a viral vector vaccine.

### Flt3L-Fc augments vaccine-induced innate immune responses and T-cell activation

**Fig 3:**
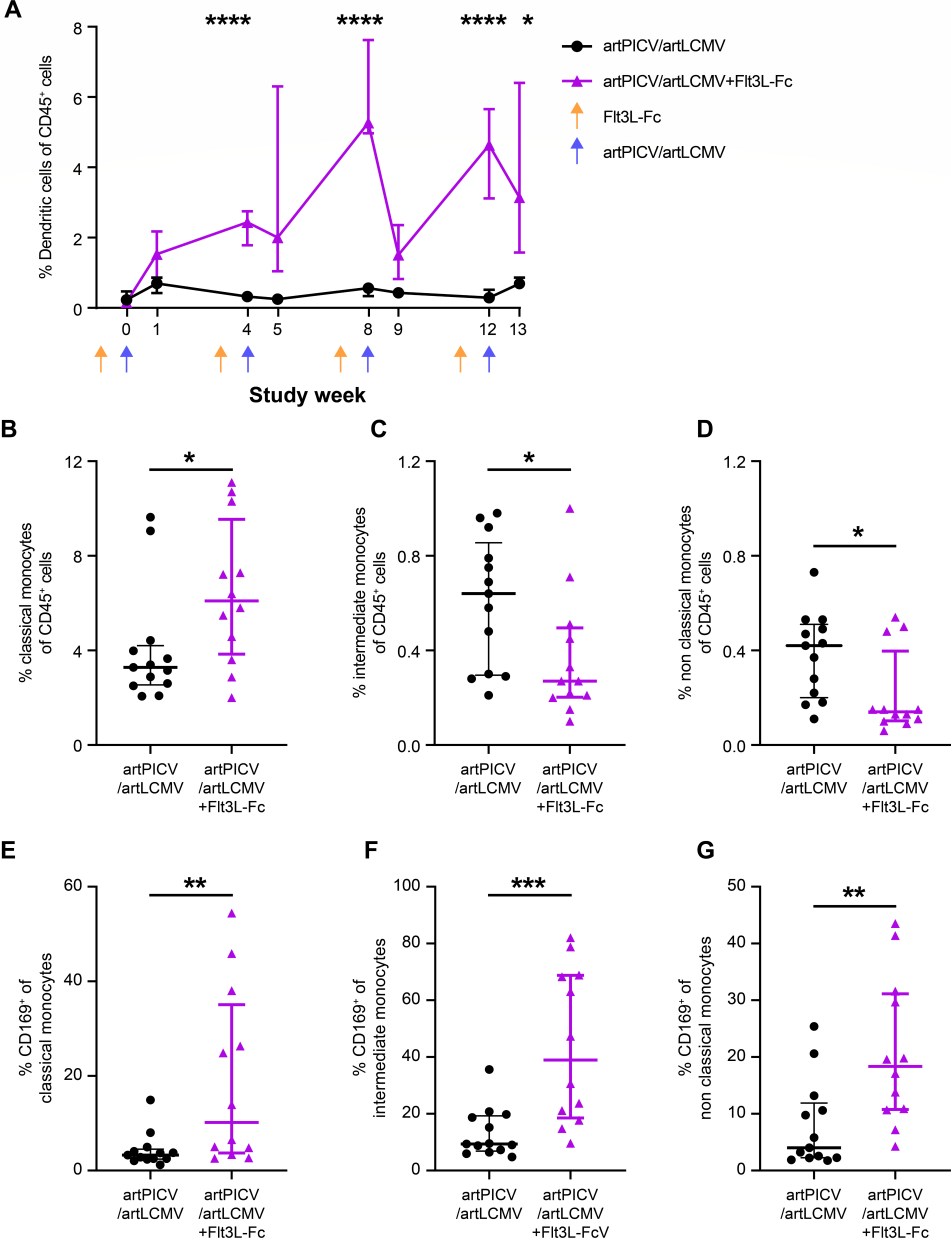
Flt3L-Fc increases peripheral dendritic cells and activates monocytes. (**A**) Frequency of dendritic cells was determined by whole blood flow cytometry on day of vaccine dosing (weeks 0, 4, 8, 12) and 1 week after vaccine dosing (weeks 1, 5, 9, 13). Dendritic cells are gated as CD45^+^CD3^–^CD8^–^CD20^–^HLA-DR^+^ CD14^–^CD16^–^CD11c^+^ cells. CD45^+^CD3^–^CD20^–^HLA-DR^+^ cells are classified into classical monocytes (CD14^+^CD16^–^), intermediate monocytes (CD14^+^CD16^+^), and nonclassical monocytes (CD14^–^CD16^+^). (B–D) Frequency of classical (**B**), intermediate (**C**), and nonclassical (**D**) monocytes at week 12. (E–G) CD169 expression on classical (**E**), intermediate (**F**), and nonclassical (**G**) monocytes at week 12. Data are median ± interquartile range (IQR). artPICV/artLCMV alone in closed circles and artPICV/artLCMV + Flt3L-Fc in closed triangles. *n* = 13/group; *P < 0.05, **P < 0.01, ***P < 0.001, ****P < 0.0001. Repeated measures two-way ANOVA with Sidak’s post-test for multiple comparisons in A, and Mann–Whitney test in B–G.

To determine the impact of Flt3L-Fc on vaccine-induced innate immune cells, we evaluated the frequency of dendritic cells and monocyte subsets in the periphery on the day of vaccine dosing (weeks 0, 4, 8, and 12) and 7 days later (weeks 1, 5, 9, and 13) by flow cytometry. We found that administration of the vaccine in combination with Flt3L-Fc led to a robust and significant increase in frequency of CD11c^+^ dendritic cells (Fig. 3A; 9.4-fold peak increase in the combination group compared with vaccine alone at week 8; *P* < 0.001). We examined the frequency of monocyte subsets at baseline and after four Flt3L-Fc doses (on week 12) by measuring CD14 and CD16 expression. We observed a significant increase in the frequency of classical monocytes (~2-fold, *P* < 0.05, Fig. 3B) and a corresponding decrease in frequency of intermediate and nonclassical monocytes (~2- to 3-fold, *P* < 0.05, Fig. 3C and D). Interestingly, all monocyte subsets show a significant increase in expression of CD169 (~4-fold, *P* < 0.01, Fig. 3E through G). CD169 expression indicates activation of monocytes for subsequent antigen cross-presentation (25).

**Fig 4:**
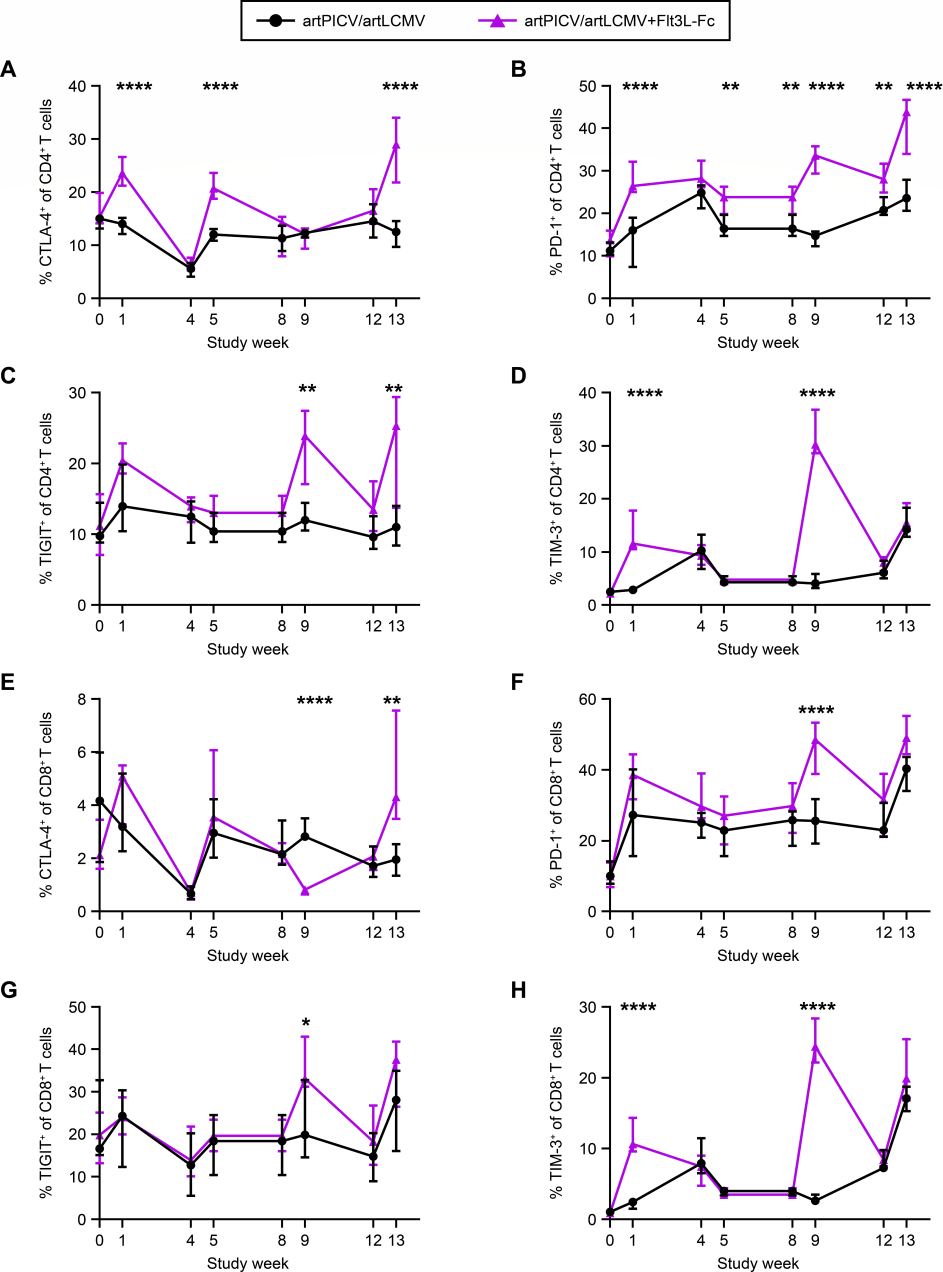
Flt3L-Fc augments vaccine-induced transient activation of CD4^+^ and CD8^+^ T cells. Frequency of expression of activation markers on CD4^+^ T cells (A–D) and CD8^+^ T cells (E–G), including CTLA-4 (in **A** and **E**), PD-1 (in **B** and **F**), TIGIT (in **C** and **G**), and TIM-3 (in **D** and **H**), was evaluated on day of vaccine dosing (weeks 0, 4, 8, and 12) and 1 week after vaccine dosing (weeks 1, 5, 9, and 13). Data are median ± interquartile range (IQR). *n*= 13/group; *P < 0.05, **P < 0.01, ***P < 0.001, ****P < 0.0001; repeated measures two-way ANOVA with Sidak’s post-test for multiple comparisons in A–H.

The increase in antigen-presenting cells led us to investigate the activation of T cells through longitudinal quantification of cytotoxic T lymphocyte antigen-4 (CTLA-4), programmed death 1 (PD-1), T-cell immunoreceptor with Ig and ITIM (immunoreceptor tyrosine-based inhibitory motif) domains (TIGIT), and T-cell immunoglobulin and mucin domain-containing protein 3 (TIM-3) expression on CD4^+^ and CD8^+^ T cells. We observed a transient but significant increase in expression of CTLA-4, PD-1, TIGIT, and TIM-3 1 week after vaccination in NHPs treated with Flt3L-Fc before vaccination as compared with vaccine alone (Fig. 4A through H). These results demonstrate the potential of Flt3L-Fc to augment vaccine-induced innate immune responses and enhance activation of CD4^+^ and CD8^+^ T cells.

### Flt3L-Fc enhances immunogenicity of arenaviral vector vaccine

To evaluate the impact of Flt3L-Fc in combination with arenavirus-based vectors to elicit SIV antigen-specific immune responses in monkeys, naïve Indian-origin rhesus monkeys were immunized via the intramuscular route with artPICV and artLCMV vectors expressing SIV_SME543_ Gag, Env, and Pol immunogens either alone or in combination with Flt3L-Fc (*n* = 13/group; Fig. 2A). Administration of the vectors and Flt3L-Fc was found to be safe and well tolerated, with no treatment-related adverse events or safety concerns appearing during the study.

**Fig 5:**
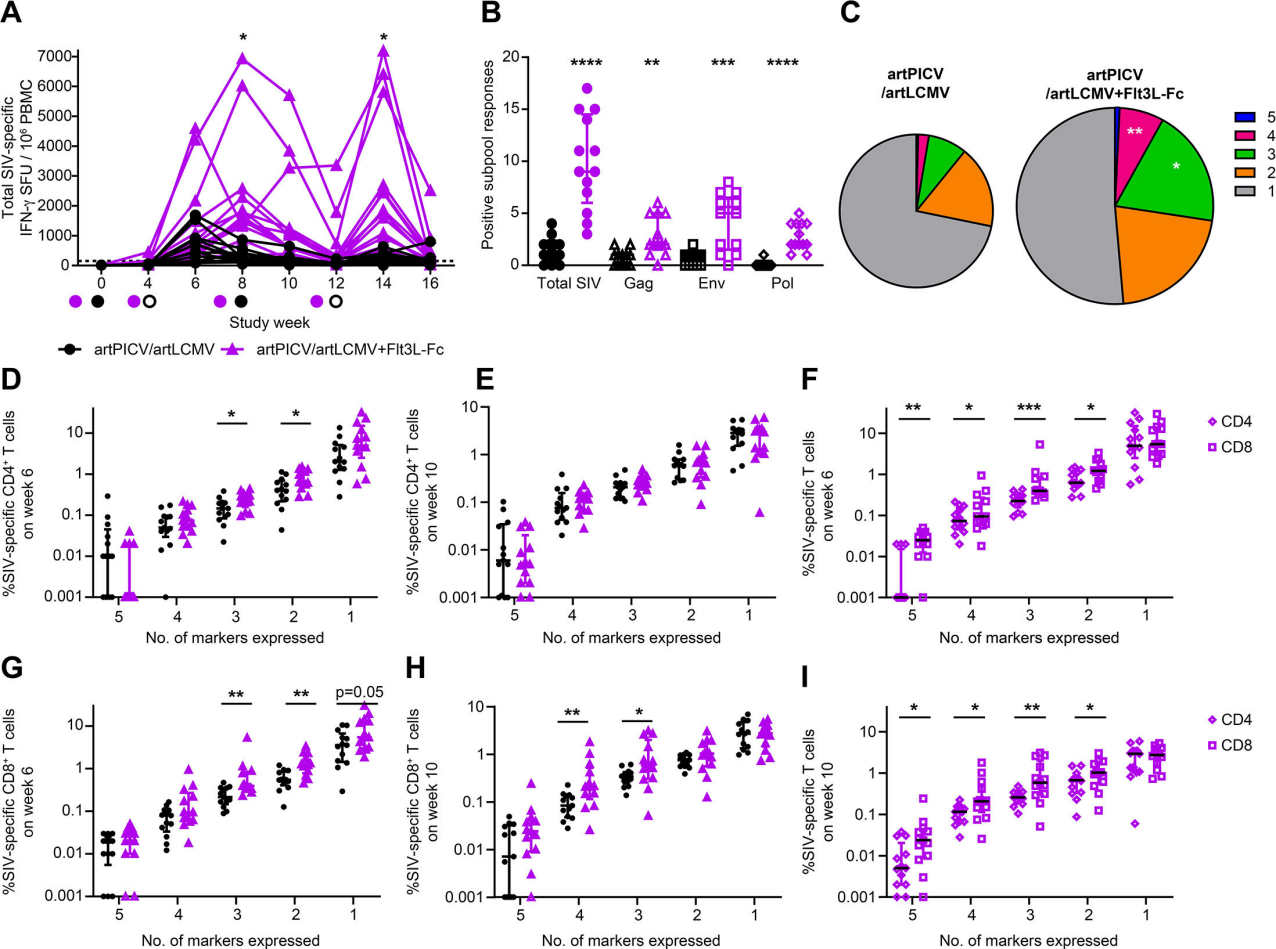
Immunogenicity of artPICV/artLCMV vaccine expressing SIV_SME543_ immunogens alone or in combination with Flt3 agonist. (**A**) Total SIV-specific IFN-γ response measured in PBMCs over weeks 0–16 of study. Circles on the x-axis indicate day of vaccination; artPICV (closed circles), artLCMV (open circles), and Flt3 agonist administration (violet closed circles). (**B**) Breadth of cellular IFN-γ responses to peptide subpools representing total SIV, Gag, Env, and Pol at week 14 for each of the 51 peptide subpools (12 for Gag, 16 for Env, and 23 for Pol). (**C**) Polyfunctional SIV-specific CD8^+^ T-cell responses expressing one to five marker combinations of IFN-γ, IL-2, TNFα, CD107a, and MIP1β at week 10 in artPICV/artLCMV alone (left) and artPICV/artLCMV in combination with Flt3L-Fc (right) with the size of the pie scaled to the total frequency of SIV-specific CD8^+^ T cells. Frequency of SIV-specific CD4^+^ and CD8^+^ T cells at week 6 (**D and G**) and week 10 (**E and H**). Comparison of SIV-specific CD4^+^ and CD8^+^ T-cell responses in NHPs that received artPICV/artLCMV and Flt3L-Fc at week 6 (**F**) and week 10 (**I**). Data are median ± interquartile range (IQR); *n* = 13/group; *P < 0.05, **P < 0.01, ***P < 0.001, ****P < 0.0001. Two-way ANOVA with Sidak’s post-test for multiple comparisons in A and C, Mann–Whitney test in B, D, E, G, and H, and Wilcoxon matched-pairs signed rank test in F and I.

We next evaluated the impact of Flt3L-Fc on immunogenicity of the artPICV/artLCMV vaccine vectors through measurements of cellular and humoral immune responses. Administration of Flt3L-Fc before vaccination stimulated significantly stronger Gag-, Env-, and Pol-specific cellular responses over vaccination alone, as determined by IFN-γ enzyme-linked immunosorbent spot (ELISpot); median seven- to eight-fold increases were noted after boosts at doses 3 and 4 (Fig. 5A). We also evaluated the generation of artPICV and artLCMV vector nucleoprotein (NP)-specific cellular immune responses by vector NP-specific IFN-γ response ELISpot. The addition of Flt3L-Fc dosing increased the NP-specific responses. However, these NP-specific responses were significantly lower than SIV-specific IFN-γ responses after boosting at matched time points in both groups, demonstrating that NP-specific responses did not impact SIV-specific immunity (Fig. S4). Repeated dosing with Flt3L-Fc in combination with vaccination significantly enhanced the cellular breadth compared with vaccine alone when measuring the IFN-γ ELISpot response to 51 SIV peptide subpools, comprising Gag (12 subpools), Env (16 subpools), and Pol (23 subpools). We found that after the four vaccine doses, the median SIV breadth in the Flt3L-Fc-treated animals was to nine subpools out of the 51. By comparison, the median breadth in the vaccine-alone group was to one subpool. Therefore, Flt3L-Fc treatment before vaccination led to a nine-fold increase in breadth of response (*P* < 0.001, Fig. 5B). Flt3L-Fc treatment in combination with vaccination also led to a two- to three-fold increase in autologous and heterologous gp120 Env binding Ab titers (SIV_SME543_ and SIV_MAC251_) (*P* < 0.05, Fig. S5). As studies have shown that Flt3L-Flt3 signaling can induce suppressive CD4^+^ regulatory T cells (Tregs) (26), we evaluated the kinetics of Tregs in the periphery. We observed a small but significant increase in Tregs after the first and third doses of Flt3L-Fc. Importantly, no difference in Treg levels was observed after all four doses of Flt3L-Fc were administered when compared with vaccine alone (Fig. S6A), and the presence of Tregs does not impact the magnitude of SIV-specific cellular responses (Fig. S6B).

SIV-specific T-cell responses were characterized for effector functions, including secretion of IFN-γ, IL-2, tumor necrosis factor (TNF)-α, and expression of MIP1β and CD107a by multiparameter intracellular flow cytometry staining. Polyfunctionality of SIV-specific T-cell responses was characterized at baseline and 2 weeks after the second and third vaccine doses (weeks 6 and 10). No significant differences were observed at baseline between the two groups. The frequency of SIV-specific CD4^+^ and CD8^+^ T cells expressing any two, three, four, or all five of the markers (IFN-γ, IL-2, TNF-α, MIP1β, and CD107a) was evaluated through Boolean gating of SIV-specific T-cell populations. Polyfunctional SIV-specific CD4^+^ T cells were detected at week 6 in both the combination and the vaccine-alone groups, with the Flt3L-Fc-treated group having significantly higher percentages of bifunctional and trifunctional T cells over vaccine alone (*P* < 0.05, Fig. 5D). However, by week 10, there were no significant differences in the frequency of SIV-specific polyfunctional CD4^+^ T-cell responses (Fig. 5E). CD8^+^ T cells at week 6 also showed a significant increase in bifunctional and trifunctional SIV-specific responses in animals treated with Flt3-Fc before vaccination (*P* < 0.01, Fig. 5G). Polyfunctional CD8^+^ T-cell responses remained significantly higher at week 10 with Flt3L-Fc treatment for trifunctional and tetrafunctional cell populations, indicative of sustained maintenance of polyfunctional SIV-specific CD8^+^ T-cell responses over time. Taken together, the animals that received the Flt3L-Fc before vaccination stimulated peak SIV-specific polyfunctional CD8^+^ T-cell responses after the third vaccine dose (Fig. 5C). The increase in SIV-specific polyfunctional T-cell responses in the animals that received Flt3L-Fc before vaccination led us to compare the proportion of polyfunctional CD4^+^ and CD8^+^ T-cell responses at week 6 and week 10 in these animals. Interestingly, the polyfunctional response was significantly higher in CD8^+^ T cells than in CD4^+^ T cells after the second and third vaccine doses with four- to five-fold higher frequency of SIV-specific CD8^+^ T cells expressing all five functional markers than the corresponding SIV-specific CD4^+^ T cells (Fig. 5F and I). These results suggest the potential of Flt3L-Fc to promote more polyfunctional CD8^+^ than CD4^+^ T-cell response.

### Comparative immunogenicity analysis of Flt3L-Fc and checkpoint inhibitors

**Fig 6:**
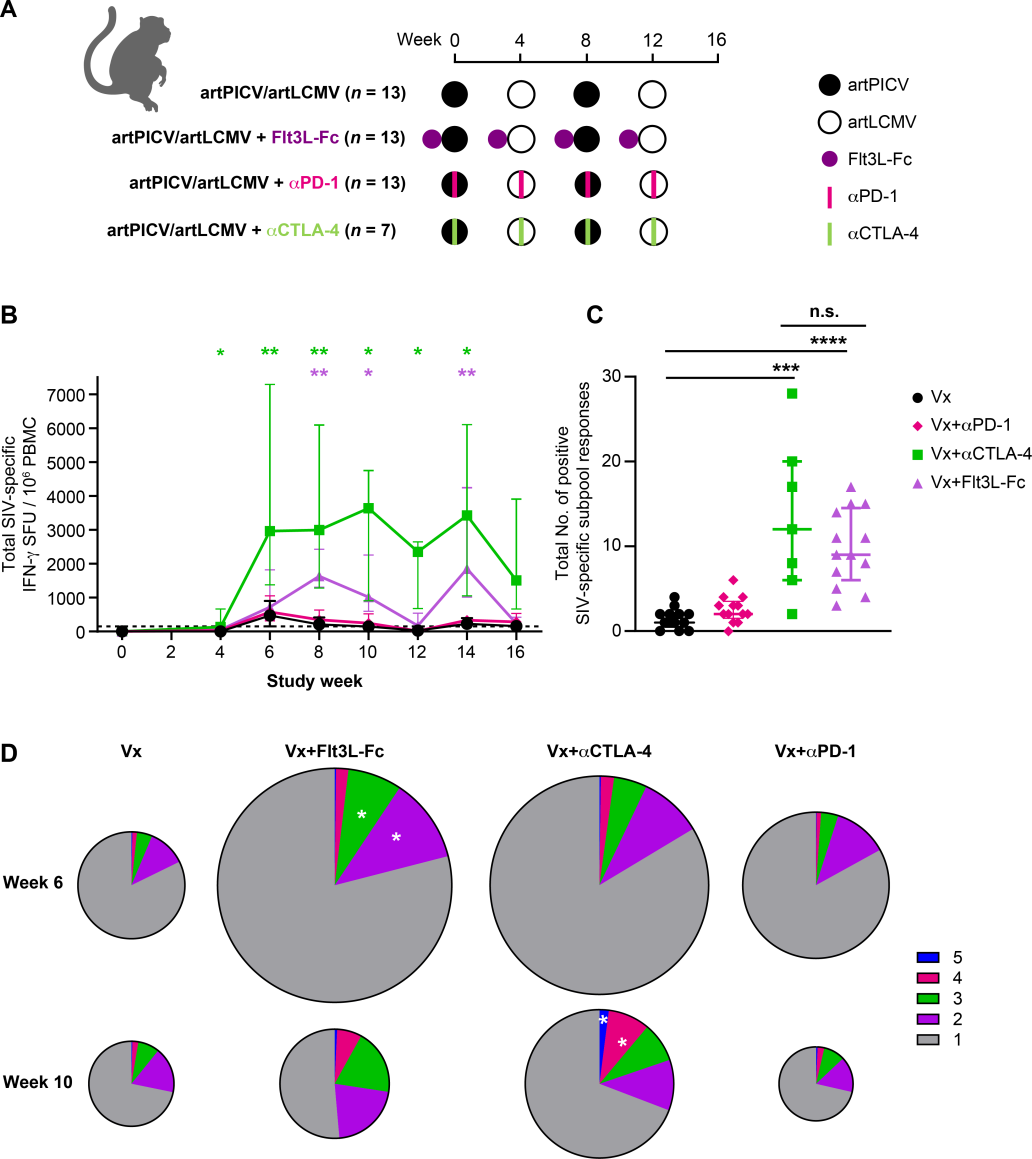
Immunogenicity of artPICV/artLCMV vaccine expressing SIV_SME543_ immunogens alone or in combination with Flt3 agonist or checkpoint inhibitors. (**A**) Schematic of NHP study design. (**B**) Total SIV-specific IFN-γ response measured in PBMCs over weeks 0–16 of study. (**C**) Breadth of cellular IFN-γ responses to peptide subpools representing total SIV, Gag, Env, and Pol at week 14 for each of the 51 peptide subpools (12 for Gag, 16 for Env, and 23 for Pol). (**D**) Polyfunctional SIV-specific CD8^+^ T-cell responses at week 6 (top) and week 10 (bottom) expressing one to five marker combinations of IFN-γ, IL-2, TNF-α, CD107a, and MIP1β with the size of the pie scaled to the total frequency of SIV-specific CD8^+^ T cells. Data are median ± interquartile range (IQR); *n* = 7/group in all groups except in combination with αCTLA-4 (*n* = 7); *P < 0.05, **P < 0.01, ***P < 0.001, ****P < 0.0001, n.s., not significant. Two-way ANOVA with Sidak’s post-test for multiple comparisons in B, Friedman’s one-way ANOVA with Dunn’s post-test in C, and Mann–Whitney test comparison of all groups to artPICV/artLCMV group with Benjamini Hochberg correction for multiple testing in D.

Checkpoint inhibitors such as αPD-1 and αCTLA-4 have been shown to enhance immunogenicity of viral vaccine vectors in NHPs (27, 28) and are currently being investigated in several clinical trials in oncology (29, 30). This led us to evaluate the immunogenicity of the arenavirus SIV vaccine in combination with αPD-1 or αCTLA-4 (Fig. 6A). The serum PK of αPD-1 and αCTLA-4 were evaluated, and comparable C_max_ was observed after each dose (Fig. S7A and B). The plasma exposure of the αPD-1 blocking antibody is comparable in this study to its use in prior studies in rhesus monkeys (31). Unlike the robust increase in DCs and monocytes observed in NHPs administered vaccine after administration of Flt3L-Fc, no change in DCs and monocytes was observed in NHPs that received the vaccine in combination with αPD-1 or αCTLA-4 (Fig. S8A and B). We evaluated the impact of immune modulation on vaccine immunogenicity by measuring cellular and humoral immune responses. Combination of the vaccine with Flt3L-Fc or αCTLA-4 but not αPD-1 resulted in strongly enhanced Gag-, Env-, and Pol-specific cellular responses as determined by IFN-γ ELISpot (peak fold increase at week 14: eight-fold with Flt3L-Fc and 14-fold with αCTLA-4) compared with animals that received the vaccine alone (Fig. 6B). The breadth of cellular responses at week 14 was also significantly increased when vaccine is administered with Flt3L-Fc (nine-fold) or αCTLA-4 (12-fold), and remained unchanged when co-dosed with αPD-1 when compared with vaccine alone (Fig. 6C). Of note, the magnitude and breadth of SIV-specific IFN-γ responses were not significantly different between animals that were administered vaccine in combination with Flt3L-Fc or αCTLA-4 (Fig. 6B and C). Polyfunctional SIV-specific CD4^+^ T-cell responses were comparable between all the groups at weeks 6 and 10. Interestingly, combination with αCTLA-4 resulted in SIV-specific polyfunctional CD8^+^ T-cell responses that were significantly higher than vaccine alone by week 10 and comparable with animals that received Flt3L-Fc before vaccination (Fig. 6D). Combination of vaccine with αPD-1 did not enhance the magnitude, breadth, and polyfunctionality of T-cell responses compared with vaccine alone (Fig. 6B through D). These results demonstrate that the enhancement of arenaviral vector vaccine immunogenicity is comparable between Flt3L-Fc and αCTLA-4 with differences in induction of innate immune cells. Binding of checkpoint inhibitor αCTLA-4 to CTLA-4 on T cells modulates T-cell activation, whereas Flt3L-Flt3 interaction on DCs leads to increased DC maturation and antigen presentation. The comparable immunogenicity of arenaviral vectors in combination with Flt3L-Fc or αCTLA-4 is interesting given the different mechanisms of action of the two immune modulators, providing new modalities for augmenting vaccine responses.

## DISCUSSION

Flt3L is a hematopoietic growth factor that binds to Flt3, a tyrosine kinase receptor, on myeloid and lymphoid progenitor cells in the bone marrow and terminally differentiated DCs (32), leading to an expansion of cDC1s in the periphery. Multiple studies in cancer immunotherapy are investigating strategies to expand cDC1 in the tumor through use of a soluble recombinant Flt3 protein, CDX-301. In a phase 1 clinical trial in healthy volunteers, daily subcutaneous administration of CDX-301 for 5–10 days resulted in robust expansion of cDC1s and monocytes in the periphery (22). GS-3583 has been engineered as a human Flt3L-Fc fusion protein with longer half-life to evaluate the impact of Flt3 signaling in modulating antitumor T-cell immunity. GS-3583 demonstrated safety, tolerability, and dose-dependent expansion of DCs in the periphery in healthy volunteers (33). In a phase 1b study of participants with advanced solid tumors, administration of GS-3583 led to robust expansion of DCs in the periphery with a trend toward dose-dependent DC durability (34). Published studies show that arenaviral vectors exhibit increased tropism to myeloid DCs and monocytes leading to activation of myeloid DCs (13, 35). The induction of DCs by Flt3 ligand and increased uptake of arenaviral vectors by myeloid DCs led us to hypothesize that systemic administration of Flt3L-Fc before arenaviral vaccine dosing could enhance vaccine-induced T-cell responses.

To test our hypothesis that Flt3L will significantly increase SIV vaccine responses when using an arenaviral vector platform, we engineered a rhesus Flt3L-Fc fusion molecule. This molecule, comprising rhesus fms-related tyrosine kinase 3 ligand (Flt3 ligand, Flt3L) extracellular domain fused to the fragment crystallizable (Fc) region of rhesus IgG1, allowed us to examine Flt3L-Flt3 biology without the need for daily dosing in rhesus macaques. Through a dose-finding study in healthy rhesus, the dose of 0.3 mg/kg of Flt3L-Fc was chosen based on robust expansion of cDC1 and cDC2 at 7 days after dosing relative to the other doses tested and utilized in subsequent combination study with arenaviral vectors. Combination of Flt3L-Fc with arenaviral vaccine encoding SIV immunogens led to increases in frequency and activation of cDC1s and increases in monocytes in whole blood count assay, without a sustained increase in CD4^+^ Tregs. Published studies have shown that Flt3L can induce major histocompatibility complex II expression on bone marrow hematopoietic cell-derived cDCs *in vitro* and on plasmacytoid DCs when administered in combination with an RNA vaccine in mice, indicating that Flt3L can enhance activation and maturation of DCs (36, 37). Our results are in line with published studies that demonstrate a soluble recombinant Flt3 protein induces a significant increase in DCs and monocytes (22). We further evaluated the impact of Flt3L-Fc on monocyte subsets and observed an increase in frequency of CD169-expressing classical, intermediate, and nonclassical monocytes in animals that received Flt3L-Fc in combination with the vaccine compared with vaccine alone. CD169/Siglec-1 is expressed on the three monocyte subsets with CD169^+^ monocytes exhibiting a higher level of co-stimulatory marker expression compared with CD169^-^ monocytes. Interestingly, CD169 expression has been identified as a marker of activated monocytes with enhanced capacity for CD8^+^ T-cell activation (25). The expansion and activation of DCs and monocytes after Flt3L-Fc dosing led us to investigate the impact of innate immune-cell activation on T cells. Antigen recognition by T cells on the surface of DCs will characteristically lead to T-cell activation, proliferation, cytokine production, and effector function. Here, we show a transient increase in expression of activation markers CTLA-4, PD-1, TIGIT, and TIM3 on CD4^+^ and CD8^+^ T cells in animals that received Flt3L-Fc in combination with the vaccine, compared with the vaccine-alone group. The increase in expression of the activation markers is transient with levels returning to baseline by the next vaccine dose, indicating that repeated dosing of Flt3L-Fc and vaccine induces T-cell activation, but not exhaustion of CD4^+^ and CD8^+^ T cells.

Preclinical studies have previously demonstrated the impact of Flt3 ligand-mediated DC expansion on vaccine-induced immune responses. Systemic treatment with Flt3L before vaccination enhanced the immunogenicity and therapeutic efficacy of RNA vaccines in mouse models of melanoma (36). Similarly, pre-treatment with CDX-301 augmented therapeutic vaccine efficacy of an antigen-loaded monocyte vaccine and checkpoint inhibitor αPD-1 in a mouse model of melanoma (38). In NHPs, administration of Flt3L before DNA/MVA-vectored SIV vaccine dosed in combination with a Toll-like receptor 9 (TLR9) agonist resulted in strong SIV-specific T-cell responses and reduction in SIV viral load after SIV_MAC251_ challenge, compared with animals that received vaccine and soluble Flt3L alone, with the major effect mediated by TLR9 agonism (39). To our knowledge, studies have not evaluated the role of Flt3L-Flt3-mediated DC expansion on viral vectors with DC tropism such as arenaviral vectors. In a phase 1 study in participants with Her2/neu-overexpressing cancers, administration of Flt3 before Her2/neu-specific peptide vaccine resulted in significantly higher IFN-γ-secreting Her2/neu-specific T cells. Several ongoing and recently completed clinical trials with CDX-301, a recombinant Flt3L administered subcutaneously daily for 5–10 days, explored the potential of Flt3L-mediated DC expansion for cancer immunotherapy. In a phase 2 trial of participants with resected melanoma, daily administration of Flt3L (CDX-301) for 10 days before administration of DC-targeted vaccines led to expansion of DC subsets and significant enhancement of cellular and humoral responses (40). In line with these studies, we show that administration of a single dose of Flt3L-Fc before each vaccine dose resulted in significantly increased magnitude, breadth, and polyfunctionality of SIV-specific T-cell responses over vaccination alone. Based on these results, the potential of administering a single dose of Flt3L-Fc 7 days before vaccination could be further explored in the context of therapeutic SIV vaccine efficacy for cure studies in NHPs.

Several studies have investigated the use of checkpoint inhibitors, αCTLA-4, and αPD-1 as part of combination regimens for cancer immunotherapy and infectious diseases. After HIV infection, chronic antigen exposure leads to sustained immune activation and subsequent T-cell exhaustion, characterized by upregulation of CTLA-4 and PD-1 with increased PD-1 expression correlating to plasma HIV viremia (41, 42). Preclinical studies in macaques on PD-1 blockade have demonstrated varying levels of therapeutic efficacy depending on the stage of SIV infection and use of other agents in combination with αPD-1 (31). Although administration of αPD-1 alone did not reduce the latent reservoir in ART-suppressed people living with HIV with advanced malignancies, dosing of immune checkpoint blockade with αPD-1 and αCTLA-4 resulted in a modest decrease in markers of latent HIV reservoir in a small subset of participants in the AMC-095 study (43). In addition, studies in NHPs have evaluated the use of immune checkpoint blockade on vaccine immunogenicity. In NHPs, vaccination with adenoviral vectors encoding SIV immunogens in combination with checkpoint inhibitors such as αPD-1 increased durability of vaccine-induced T-cell responses (27), whereas combination with αCTLA-4 demonstrated an increase in magnitude of T-cell responses (28). These studies led us to compare immunogenicity of arenaviral vaccines in combination with the single-agent administration of Flt3L-Fc, αPD-1, or αCTLA-4. A significant impact of αPD-1 on vaccine-induced T-cell responses was observed when vaccination led to levels of PD-1 expression comparable with those in SIV-infected, untreated NHPs (27). In our study, there was no effect after coadministration of αPD-1 on vaccine immunogenicity, likely due to the lack of T-cell exhaustion with repeated arenaviral vaccines. Interestingly, the vaccine-induced magnitude, breadth, and polyfunctionality of T-cell responses are comparable between animals that received Flt3L-Fc or αCTLA-4. However, considering the potential safety concerns with use of αCTLA-4 observed in clinical studies (44), Flt3L-Fc demonstrates potential for further evaluation as an immune modulator to enhance vaccine-specific T-cell responses for SIV/HIV cure.

There are limitations to this study, including a lack of evaluation of SIV neutralizing antibody generation, DC subset kinetics in lymph nodes, and cellular immunogenicity in rectal tissues. Although this study demonstrates the impact of Flt3L-Fc on vaccine immunogenicity, studies in SIV-infected rhesus macaques could be performed to evaluate the benefit of Flt3L-Fc on therapeutic vaccine efficacy and identification of potential correlates of efficacy. Studies have shown the potential of combining soluble Flt3L with TLR8 agonists or αCTLA-4 to improve CD8^+^ T-cell responses in *in vitro* studies and mouse tumor models (45, 46). Such combinations could also be evaluated to understand the potential for synergism between immune modulators to augment vaccine efficacy. In conclusion, we found arenaviral vaccines and Flt3L-Fc to be safe and immunogenic with no treatment-related adverse events. Taken together, our study provides the proof-of-concept in NHPs for potential of Flt3L-Fc administration before each vaccine dose to enhance vaccine immunogenicity, and supports further development of safe and effective variants of Flt3L as part of combination therapies for HIV cure.

## MATERIALS AND METHODS

### Cloning, expression, and purification of Flt3L-Fc

Coding sequence for *Macaca fascicularis* fms-related tyrosine kinase 3 ligand isoform X14 (XP_005589969) (T27 -A178 domain) was genetically fused to N-terminus of cyno Fc harboring L234A_L235A_P331S mutation. Secretion of the FLT3L-Fc was driven by human FLT3L leader sequence. The construct was generated by gene synthesis and cloned into pcDNA3.1 expression vector (GeneArt, Regensburg, Germany). Transient transfection of Expi293 cells with the expression plasmid was conducted according to the manufacturer’s protocol. Cell-free supernatant containing expressed protein was collected 5 days post-transfection. The Flt3L-Fc was purified by a protein A affinity step followed by size exclusion chromatography on AKTA FPLC (Cytiva Life Sciences) system. Purified protein was dialyzed in 20 mM histidine pH 5.8, supplemented with sucrose/tween-80, sterile filtered, and stored at 4°C. Concentration of the formulated protein was measured by A280. Purity was assessed as percent monodispersed by analytical size-exclusion chromatography. Identity of the protein was confirmed by mass spectrometry.

### PK of Flt3L-Fc in healthy rhesus

Nine naïve adult male and female Indian-origin rhesus macaques were included in this study, with *n* = 3 per group. Animals were intravenously administered Flt3L-Fc recombinant protein at 0.1, 0.3, or 1.0 mg/kg at study days 0 and 28. The PK profile of Flt3L-Fc was evaluated through study day 56 based on serum concentration of Flt3L-Fc at 0, 0.5, and 24 hours, and 7, 14, and 28 days after each dose. The concentration of Flt3L-Fc in serum samples was determined by U-Plex Human FLT3L assay (MSD, Cat# K151XFK-2) according to the manufacturer’s instructions with minimal optimization of the method. A standard curve of Flt3L-Fc was developed, and the lower limit of quantitation (LLOQ) and upper limit of quantitation (ULOQ) were 20 and 10,000 ng/mL in 100% rhesus serum, respectively.

### Animals and vaccination

Twenty-six naïve adult (median age 3 years), both male and female, Indian-origin rhesus macaques were included in this study and maintained at the animal facility of BIOQUAL, Inc. (Rockville, MD, USA). Animals were stratified into two groups based on body weight, age, and sex with *n* = 13/group; group 1, artPICV/artLCMV vaccination alone, and group 2, artPICV/artLCMV vaccination in combination with fms-like tyrosine kinase 3 ligand-Fc (Flt3L-Fc) fusion protein. Arenavirus-based vectors, artPICV and artLCMV, were engineered to express SIV_SME543_ immunogens Gag, Env, and Pol (17). artPICV and artLCMV expressing SIV_SME543_ Gag and Env were administered in the left quadricep muscle, and the vectors expressing SIV_SME543_ Pol antigen were administered in the right quadricep muscle. At weeks 0 and 8, the animals were administered 1 × 10^6^ replication-competent virus particles (RCV) of artPICV Gag, Env, and Pol vectors. At weeks 4 and 12, the animals were administered 4 × 10^6^ RCV of artLCMV Gag and Env vectors, and 2 × 10^6^ RCV of artLCMV Pol vectors. The animals in group 2 were administered four total doses of Flt3L-Fc recombinant protein at 0.3 mg/kg via intravenous injection 7 days before each vaccination. An additional 20 naïve adult animals were added to the study to evaluate the impact of checkpoint inhibition. Thirteen NHPs in group 3 were administered αPD-1 intravenously at weeks 0, 4, 8, and 12 of study at 10 mg/kg at the same time as the vaccine vectors above. Seven NHPs in group 4 were administered αCTLA-4 intravenously at weeks 0, 4, 8, and 12 of study at 10 mg/kg at the same time as the vaccine vectors above.

### PK of αPD-1 in rhesus

A Meso Scale Discovery (MSD) electrochemiluminescence (ECL)-based detection method was developed for determination of αPD-1 chimeric rhesus IgG4 antibody in serum of healthy rhesus macaques. Briefly, αPD-1 in serum was captured on small-spot streptavidin MSD plates coated with biotin-conjugated recombinant human PD-1 protein followed by detection with ruthenylated antihuman/NHP antibody. The concentration of αPD-1 in samples was then determined based on the produced ECL signals, which is proportional to the amount of αPD-1 bound to the plate from a standard curve using a four-parameter logistic curve fit. The LLOQ and ULOQ were 100 and 5,000 ng/mL in 100% rhesus serum, respectively.

### PK of αCTLA-4 in rhesus

The concentration of αCTLA-4 (ipilimumab) in rhesus serum was determined using MSD ECL-based immunoassay platform. Briefly, αCTLA-4 was captured by the recombinant human CTLA-4 protein coated on standard MSD plates followed by detection with goat antihuman IgG antibody. The amount of bound αCTLA-4 in the samples was detected based on the amount of the light generated and measured by the MSD plate reader. A four-parameter logistic curve fit of an established standard curve was used to quantitate αCTLA-4 in samples. The LLOQ and ULOQ were 3 and 3,000 ng/mL in 100% rhesus serum, respectively.

### AML5 cell proliferation assay

AML5 cells (DSMZ, Germany) were cultured in MEMα media containing 20% heat-inactivated fetal bovine serum. The cells were cultured in serum-free media overnight before addition of Flt3L-Fc. To obtain a 12-point dose–response curve, Flt3L-Fc or the control isotype 1 was added at a four-fold serial dilution starting at 10 nM. The cells were cultured for 72 hours, and cell proliferation was evaluated by CellTiterGlo assay, following the manufacturer’s instructions. Briefly, 50 µL of CellTiterGlo reagent was added, incubated at room temperature (RT) for 10 minutes, and the resulting luminescence signal was read using an EnVision plate reader (PerkinElmer, Waltham, MA, USA). Assay signals were plotted, and dose–response curves were generated in GraphPad Prism v8.1.2 to determine EC_50_ values.

### IFN-γ ELISpot

We determined T-cell responses to SIV peptide stimulation through IFN-γ ELISpot. The peripheral blood mononuclear cells (PBMCs) were isolated from 5–10 mL of whole blood by density gradient centrifugation. ELISpot plates precoated with an anti-IFN-γ capture antibody (ELISpot Plus: Monkey IFN-γ [horseradish peroxidase {HRP}], Mabtech AB, Nacka Strand, Sweden), and ELISpot testing was performed on freshly isolated samples according to the manufacturer’s instructions. Peptide pools for stimulation included SIV_SME543_ Gag, Env, Pol, and vector-specific NP (LCMV and PICV) added at a final peptide concentration of 1 µg/mL. Phytohemagglutinin at a final concentration of 5 µg/mL served as a positive control. Dimethyl sulfoxide (DMSO) served as a negative control at a final concentration identical to the DMSO in the peptide stimulations. For measurement of cellular immune breadth, SIV_SME543_ Gag, Env, and Pol subpools were used (10 peptides/pool; 51 pools in total, including 12 for Gag, 16 for Env, and 23 for Pol; each peptide was a 15-mer with an 11 amino-acid overlap). The plates were incubated at 37°C, 5% CO_2_ for 20–24 hours before development of IFN-γ spots. The spots were visualized through a two-step binding process using an anti-IFN-γ–biotin detection antibody, followed by a tertiary streptavidin-HRP; IFN-γ spot-forming units were visualized using a chromogenic HRP substrate.

### Intracellular cytokine staining multiparameter flow cytometry

Intracellular staining for flow cytometry was performed using predetermined concentrations of antibodies per the manufacturer’s recommendations for CD3 (Alexa Fluor 700, SP34-2, BD Biosciences, Franklin Lakes, NJ, USA), CD4 (OKT4, Brilliant Violet 605, BioLegend, Inc., San Diego, CA, USA), CD8 (RPA-T8, Brilliant Violet 650, BioLegend), IFN-γ (B27, PE-CF594, BD Biosciences), IL-2 (MQ1-17H12, PE, BD Biosciences), TNF-α (MAB11, PerCP-Cy 5.5, BD Biosciences), CD107a (H4A3, APC, BioLegend), and MIP1β (D21-1351, BD Biosciences, 560565). Freshly isolated PBMCs were plated at 500,000 cells/well in 96-well plate and were stimulated with SIV peptide pools at 2 µg/mL, phorbol-12-myristate-13-acetate (50 ng/mL)/ionomycin (500 ng/mL), or DMSO control. Cells were stained for cell surface markers followed by membrane permeabilization (eBioscience Foxp3/Transcription Factor Staining Buffer Set [catalog number 00-5523-00], Invitrogen/Thermo Fisher) to stain for intracellular markers. Samples were collected on BD LSRFortessa, and data were analyzed using FlowJo v10.7.1 (BD, Ashton, OR, USA) by the gating strategy outlined in Boopathy et al. (17) and in Fig. S9.

### Whole blood multicolor flow cytometry

Whole blood was plated at 100 µL/well and was centrifuged at 500 × *g* for 5 minutes in MACS running buffer followed by wash with PBS. Cells were resuspended in buffer containing live/dead stain (FVS780, BD Biosciences) and were incubated for 20 minutes at RT in the dark. Cells were centrifuged at 500 × *g* for 5 minutes then resuspended in prepared Fc Block and centrifuged again. For evaluation of T-cell activation, the following antibody master mix containing antibodies for CCR7 (G043H7, AF700, BioLegend), CD3 (HIT3a, BV605, BD Biosciences), CD4 (RPA-T4, BV421, BioLegend), CD8 (53-6.7, BV570, BioLegend), PD-1 (29F.1A12, PE-Cy7, BioLegend), TIGIT (MBSA43, PE-eFlour 610, Thermo Fisher), CD45RA (L48, FITC, BD Biosciences), Tim-3 (7D3, BV650, BD Biosciences), and CD69 (FN50, BB700, BD Biosciences) was added to the cells and incubated for 40 minutes at RT in the dark. For evaluation of DC and monocytes, cells were mixed with antibody master mix containing antibodies for CD3 (SK7, APC-Cy7, BD Biosciences), CD8 (53-6.7, BV570, BioLegend), HLA-DR (L243, BV421, BioLegend), NKG2A (Z199, APC, Beckman Coulter), CD11c (3.9, PE/Dazzle594, BioLegend), CD14 (M5E2, AF488, BD Biosciences), CD20 (L27, BV605, BD Biosciences), CD69 (FN50, BB700, BD Biosciences), CD86 (GL1, BV650, BD Biosciences), CD16 (3G8, BV510, BD Biosciences), CD169 (7-239, PE, BD Biosciences), and CD45 (HI30, PE-Cy7, BD Biosciences), and were incubated for 40 minutes at RT in the dark. The stained cell suspension was incubated for 20 minutes at RT with lysis buffer and was centrifuged at 500 *× g* for 5 minutes. Samples were collected on MACS Quant 16 Flow Cytometer, and data were analyzed by FlowJo v10.7.1 (BD).

### Quantification of CD4^+^ Tregs

Whole blood was plated at 100 µL/well and centrifuged at 500 *× g* for 5 minutes in MACS running buffer followed by wash with PBS. Cells were resuspended in buffer containing live/dead stain (FVS780, BD Biosciences) and were incubated for 20 minutes at RT in the dark. Cells were centrifuged at 500 *× g* for 5 minutes then resuspended in prepared Fc Block (TruStain FcX, BioLegend) and were centrifuged again at 500 *× g* for 5 minutes. For evaluation of Tregs, cells were mixed with the following antibody master mix containing antibodies for CCR7 (G043H7, AF700, BioLegend), CD3 (HIT3a, BV605, BD Biosciences), CD4 (RPA-T4, BV421, BioLegend), CD8 (53–6.7, BV570, BioLegend), PD-1 (29F.1A12, PE-Cy7, BioLegend), CD25 (BC96, APC, BioLegend), CD45RA (L48, FITC, BD Biosciences), HLA-DR (L243, BV510, BioLegend), CD127 (A7R34, PerCP-Cy5.5, Thermo Fisher), CD28 (CD28.2, BV650, BioLegend), and CTLA-4 (UC10-4F10-11, PE-CF594, BD Biosciences), and were incubated for 40 minutes at RT in the dark. The stained cell suspension was then incubated for 20 minutes at RT with lysis buffer and was centrifuged. Cells were then incubated for 10 minutes at 4°C in 1× permeabilization buffer. Cells were centrifuged and resuspended in an intracellular staining antibody mix containing antibodies for CTLA-4 (UC10-4F10-11, PE-CF594, BD Biosciences) and FoxP3 (FJK-16s, PE, Thermo Fisher), and were incubated for 40 minutes at RT in the dark. Cells were washed and resuspended, samples were collected on MACS Quant 16 Flow Cytometer, and data were analyzed by FlowJo v10.7.1 (BD).

### Quantification of cDC1 in peripheral blood

Whole blood samples (300 µL) were transferred to TruCount tubes and incubated with 15 µL of TruStain FcX Block for 15 minutes at RT. Samples were incubated with primary antibodies for CD123 (7G3, BD Biosciences, 560087), CD80 (L307.4, BD Biosciences, 565157), HLA-DR (L243, BioLegend, 307618), CD16 (3G8, BioLegend, 302038), CD20 (2H7, BD Biosciences, 563067, dump channel), CD56 (NCAM16.2, BD Biosciences, 563041, dump channel), CD3 (SP34-2, BD Biosciences, 560770), CD86 (FUN-1, BD Biosciences, 563412), CD1c (L161, BioLegend, 331536), CD11c (3.9, BioLegend, 301644), CD141 (1A4, BioLegend, 565084), Clec9A (9A11, Invitrogen, 46-3709-42), CD135 (BV10A4H2, BioLegend, 313306), and CD14 (M5E2, BioLegend, 301814) based on the manufacturer’s instructions and pilot titration assays. After 30 minutes at RT, cells were incubated with BDPharm Lyse lysing solution for 8 minutes at RT. Cells were pelleted and incubated with Live/Dead fixable Aqua stain (Thermo Fisher Scientific) followed by centrifugation and incubation in fixation buffer (Thermo Fisher Scientific) for 20 minutes on ice before acquisition on the BD Fortessa flow cytometer and gating strategy outlined in Fig. S10.

### Env IgG enzyme-linked immunoassay

Detection of IgG antibodies to Env was conducted through enzyme-linked immunoassay. Thermo Scientific Nunc MaxiSorp 384 well assay plates (Thermo Fisher) were coated overnight at 4°C with SIV gp120 recombinant proteins (2 µg/mL). Plates were washed three times with phosphate buffered saline (PBS) with Tween pH 7.4 containing 0.05% Tween 20 (PBST) and were blocked with Dulbecco’s PBS pH 7.4 containing 5% skim milk and 1% goat serum for 1 hour at RT. Blocking reagent was then aspirated, and 25 µL of an eight-point, three-fold serial dilution (1:15 starting dilution, followed by 1:3 dilution) of heat-inactivated sera prepared in diluent (Dulbecco’s Modified Eagle Medium containing 2% fetal bovine serum) was added to the plates. Pooled sera from three naïve NHPs were diluted similarly to the test sera and used as negative control. Plates were incubated for 1 hour at 4°C, followed by three washes with PBST. Immediately after washing, 25 µL of goat anti-monkey IgG (H+L)-HRP secondary antibody diluted 1:100,000 in Dulbecco’s PBS pH 7.4 containing 1% bovine serum albumin was added to each well of the plate and was incubated for 30 minutes at RT. Plates were then washed three times with PBST, 25 µL of tetramethylbenzidine substrate was added, incubated at RT for 20 minutes, and the reaction was quenched with 25 µL of 0.16 M sulfuric acid. The absorbance of the plates was read at 450 nm on EnVision XCite multimode plate reader (PerkinElmer). Duplicate A_450_ negative control values (naïve NHP sera) were averaged. Endpoint titers were reported as the highest dilution of serum samples with average A_450_ values that were three standard deviations above the negative controls.

### Statistical analysis

Immunologic data from the study were analyzed using GraphPad Prism 8.1.2. To compare groups, two-sided Wilcoxon matched-pairs signed-rank test, Friedman’s test with Dunn’s post-test for multiple comparisons, or two-way analysis of variance (ANOVA) with Dunnett’s post-test was utilized as appropriate. Two-sided Spearman rank test was performed for correlation analyses. The experiments were not randomized, and investigators were not blinded during analyses.

## Data Availability

Gilead Sciences shares anonymized individual patient data upon request or as required by law or regulation with qualified external researchers based on submitted curriculum vitae and reflecting non-conflict of interest. The request proposal must also include a statistician. Approval of such requests is at Gilead Science’s discretion and is dependent on the nature of the request, the merit of the research proposed, the availability of the data, and the intended use of the data. Data requests should be sent to datarequest@gilead.com.

## SUPPLEMENTAL MATERIAL

Supplemental material jvi.00294-24-s0001.pdf Figures S1 to S10; Table S1.

ASM does not own the copyrights to Supplemental Material that may be linked to, or accessed through, an article. The authors have granted ASM a non-exclusive, world-wide license to publish the Supplemental Material files. Please contact the corresponding author directly for reuse.
