# Supplementary material for: Flt3 agonist enhances immunogenicity of arenavirus vector-based simian immunodeficiency virus vaccine in macaques
Source: J Virol. 2024 Jun 3;98(7):e00294-24. doi: 10.1128/jvi.00294-24 (PMC11265421; doi:10.1128/jvi.00294-24)
Supplement: Supplemental material — Figures S1 to S10; Table S1. [file jvi.00294-24-s0001.pdf]

Supplementary Figures and Tables

Flt3 agonist enhances immunogenicity of arenavirus vector-based simian immunodeficiency virus vaccine in macaques

Archana Vidya Boopathy,[1] Anurag Nekkalapudi,[1] Janette Sung,[2] Sophie Schulha,[3] Debi Jin,[4] Bhawna Sharma,[5] Sarah Ng,[6] Sabrina Lu,[4] Raphaela Wimmer,[3] Silpa Suthram,[7] Sarah Ahmadi-Erber,[3] Henning Lauterbach,[8] Klaus K Orlinger,[8] Magdeleine Hung,[4] Brian Carr,[2] Christian Callebaut,[1] Romas Geleziunas,[1] Michelle Kuhne,[6] Sarah Schmidt,[3] Brie Falkard[1#]

[1]Clinical Virology, Gilead Sciences, Inc., Foster City, California, USA. [2]Drug Metabolism, Gilead Sciences, Inc., Foster City, California, USA. [3]Virology, Hookipa Pharma Inc., New York, New York, USA. [4]Protein Therapeutics, Gilead Sciences, Inc., Foster City, California, USA. [5]Discovery Virology, Gilead Sciences, Inc., Foster City, California, USA. [6]Oncology, Gilead Sciences, Inc., Foster City, California, USA. [7]Bioinformatics, Gilead Sciences, Inc., Foster City, California, USA. [8]Global Research and Development, Hookipa Pharma Inc., New York, New York, USA.

Supplementary Figures

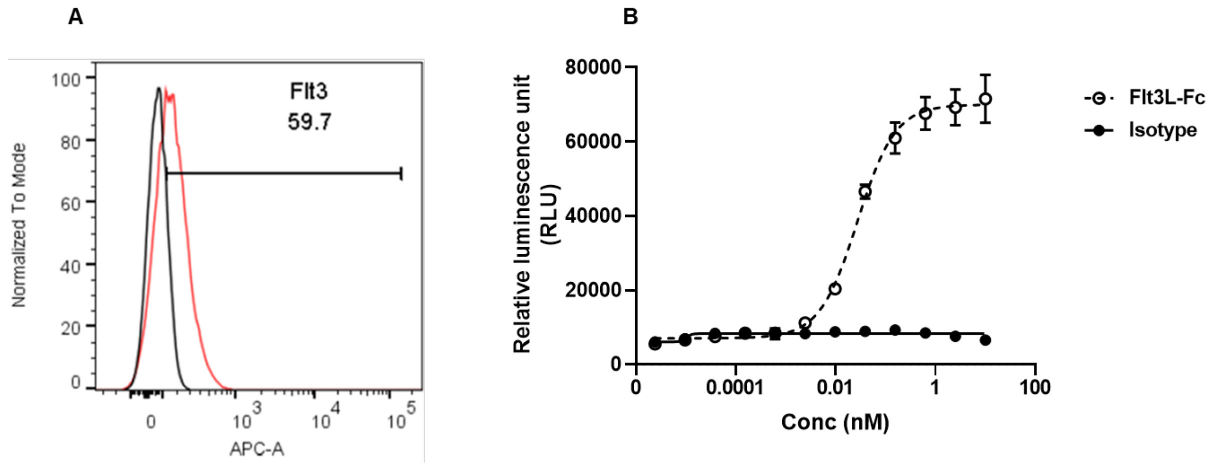

FIG S1 Flt3L-Fc dependent proliferation of AML5. (A) Flt3 expression on AML5 cells. Histogram shows AML5 cells stained with control isotype antibody (black line) and Flt3 antibody (red line). (B) Dose-dependent proliferation of AML5 cells with Flt3L-Fc (open circles) or control isotype (closed circles) after 72 hours. The y-axis, relative luminescence unit (RLU) values, reflect cell proliferation as measured by ATP Cell-Titer Glo. Symbols represent mean values and error bars represent standard deviation.

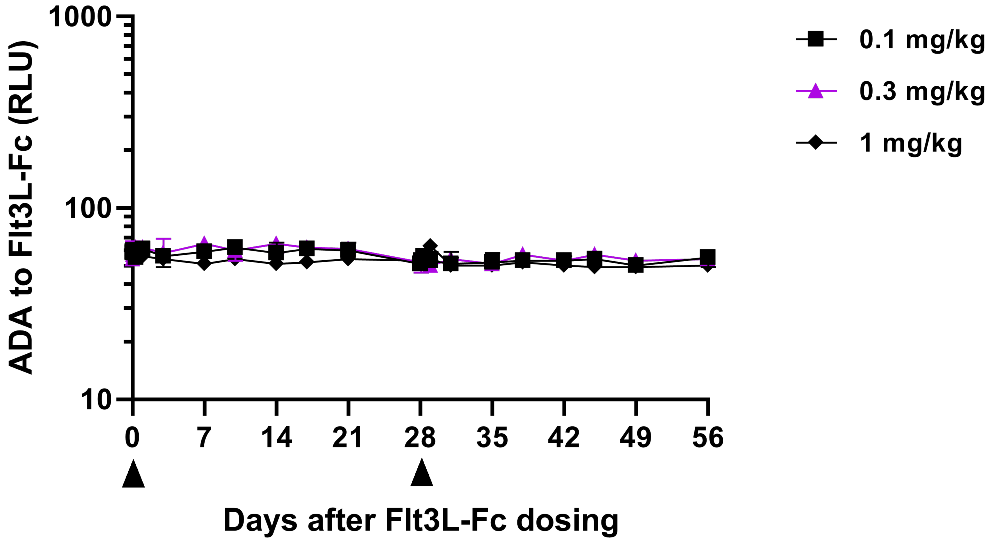

FIG S2 ADA responses to Flt3L-Fc. No antidrug antibody response was observed after two doses of Flt3L-Fc was administered at 0.1, 0.3, or 1.0 mg/kg to rhesus up to day 56 of study. Arrows on the x-axis indicate day of Flt3L-Fc dosing. Data are mean ± SD. *n* = 3/group.

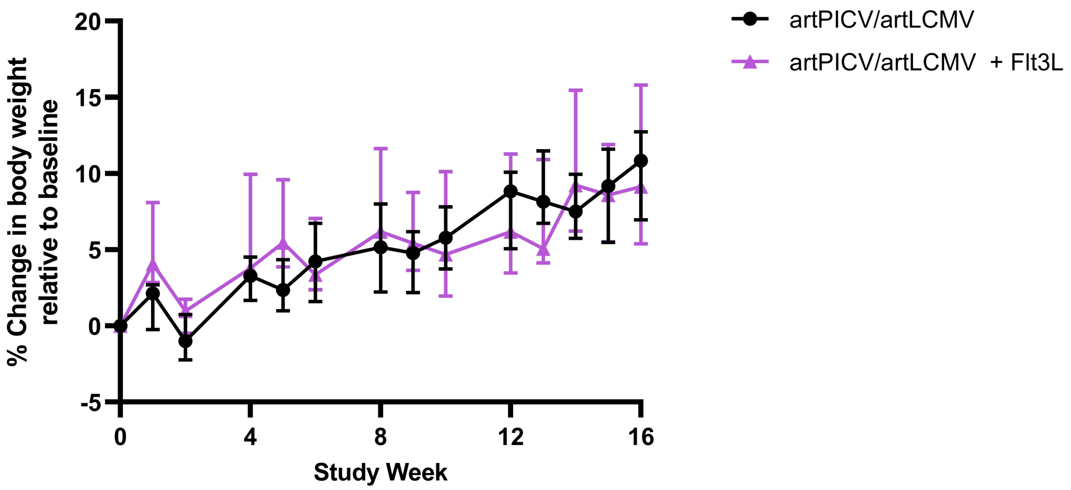

FIG S3 Body weight of NHPs over time. No significant change in body weight observed during weeks 0–16 of study. Data are median ± IQR. *n* = 13/group.

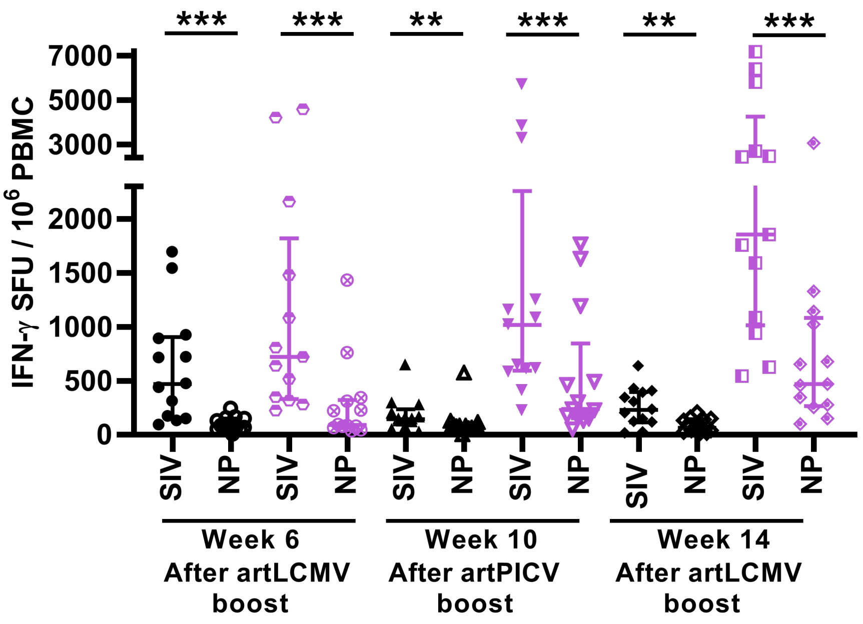

FIG S4 Induction of SIV-specific and vector-specific immune responses by artPICV/artLCMV vaccination alone or in combination with Flt3L-Fc. Peak IFN-γ responses to SIV and vector-specific NP after boost with artLCMV (week 6 and 14) and after boost with artLCMV (week 10). Data are median ± IQR. artPICV/artLCMV alone in black and artPICV/artLCMV+Flt3L-Fc in violet. *n* = 13/group. Statistical analysis by two-sided Wilcoxon matched-pairs signed-rank test. $^{**}P < 0.01$, $^{***}P < 0.001$.

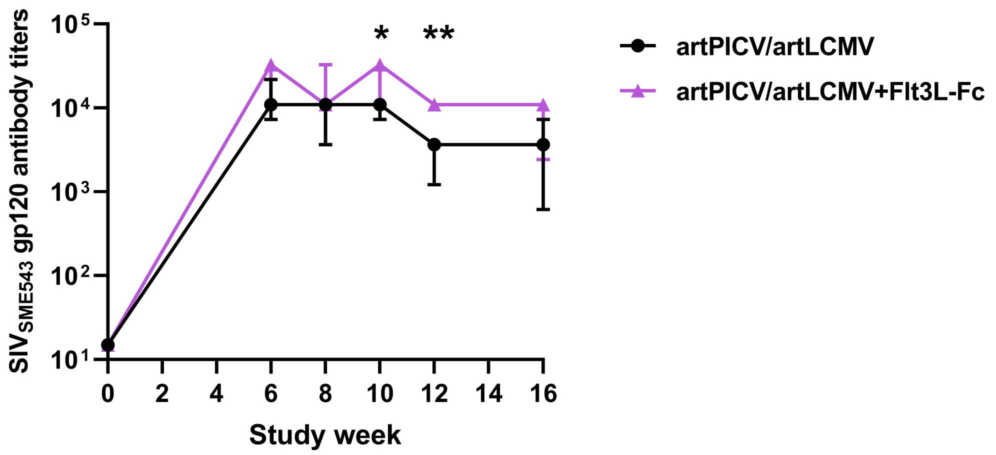

FIG S5 Kinetics of binding antibody titers to SIV_{SME543} env gp120 during weeks 0–16 of study. artPICV/artLCMV alone in black and artPICV/artLCMV+Flt3L-Fc in violet. *n* = 13/group. Data are median ± IQR. Statistical analysis by two-way ANOVA with Sidak's multiple comparison test. *$P < 0.05$, **$P < 0.01$.

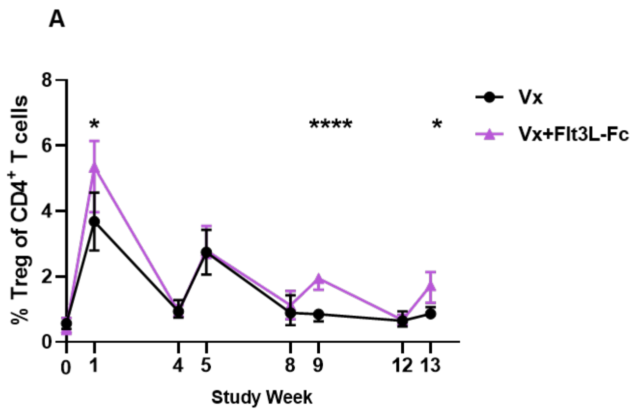

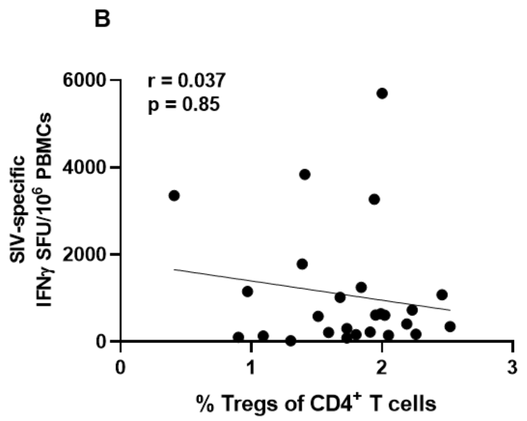

FIG S6 Kinetics of peripheral CD4$^+$ Tregs. (A) Frequency of CD4$^+$ Tregs in peripheral blood gated as CD4$^+$CD25$^+$ Foxp3$^+$CD127^{low} measured by whole blood flow cytometry during weeks 0–13 of study relative to baseline. (B) Spearman correlation of CD4 Treg frequency at weeks 9 and 13 with SIV-specific IFN-γ responses at weeks 10 and 14 of study in NHPs that received vaccine with Flt3L-Fc. *n* = 13/group. Data are median ± IQR. *$P < 0.05$, ****$P < 0.0001$, two-way ANOVA with Sidak's multiple comparison post test in (A) and nonparametric Spearman correlation in (B).

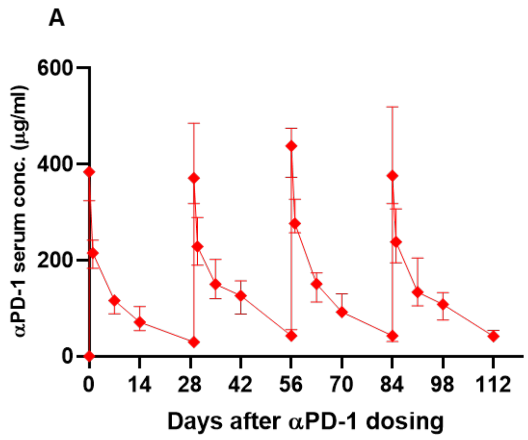

FIG S7 Pharmacokinetics of checkpoint inhibitors in NHPs administered in combination with the artPICV/artLCMV vaccine. Serum concentration of (A) αPD-1 and (B) αCTLA-4 in rhesus. *n* = 13/group. Data are median ± IQR.

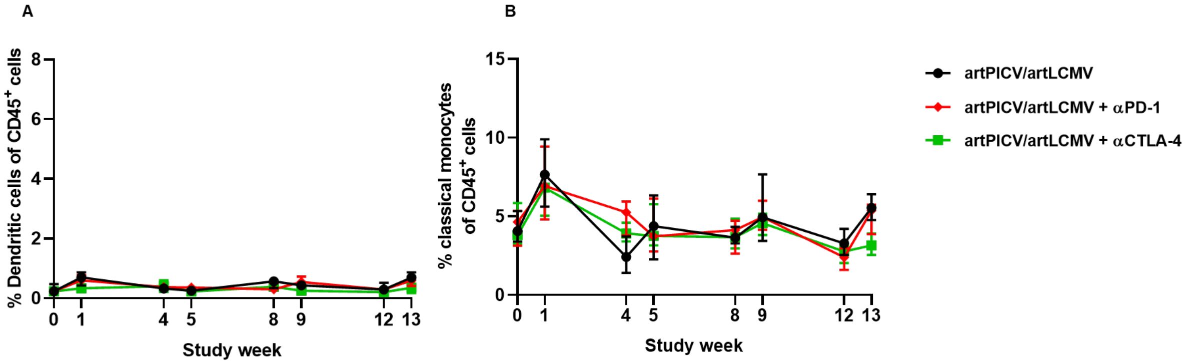

FIG S8 Checkpoint inhibitors do not impact frequency of peripheral dendritic cells and monocytes. Frequency of dendritic cells (A) and classical monocytes (B) was determined by whole blood flow cytometry on day of vaccine dosing (weeks 0, 4, 8, and 12) and 1 week after vaccine dosing (weeks 1, 5, 9, and 13). Dendritic cells are gated as $CD45^+CD3^-CD8^-CD20^-HLA\text{-}DR^+$ $CD14^-CD16^-CD11c^+$ cells. $CD45^+CD3^-CD20^-HLA\text{-}DR^+$ cells are classified into classical monocytes ($CD14^+CD16^-$). Data are median ± IQR. *n* = 13/group in groups except artPICV/artLCMV+αCTLA-4 group with *n* = 7. Two-way ANOVA with Sidak's post-test for multiple comparisons in A–B. No significant difference between groups.

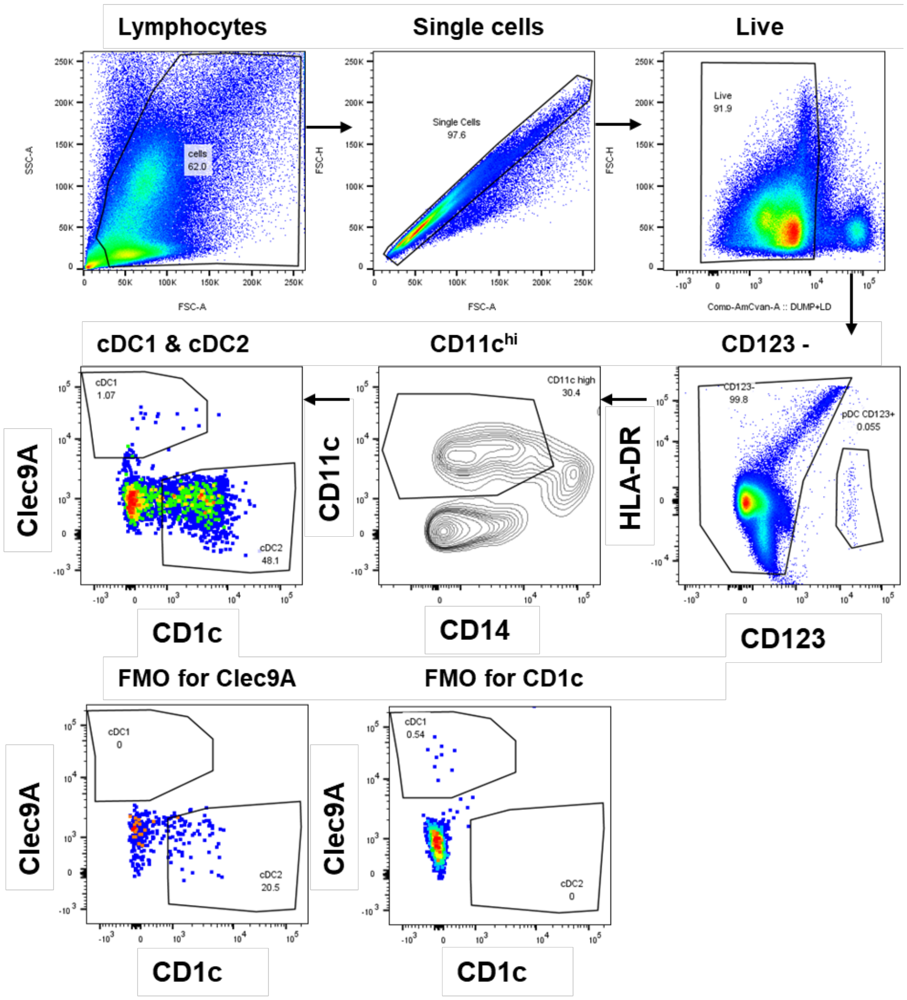

FIG S9 Flow cytometry gating strategy to identify dendritic cell subsets in peripheral blood of rhesus macaques. DC population was defined by lack of expression of lineage markers ($CD3^-CD20^-CD56^-$) and based on expression of Clec9 and CD1c as cDC1 ($CD123^-CD11c^+CD14^-Clec9^+CD1c^-$) and cDC2 ($CD123^-CD11c^+CD14^-Clec9^-CD1c^+$). FMO (fluorescence minus one) controls were run for Clec9A and CD1c to define the gates for cDC1 and cDC2, as shown in the bottom row.

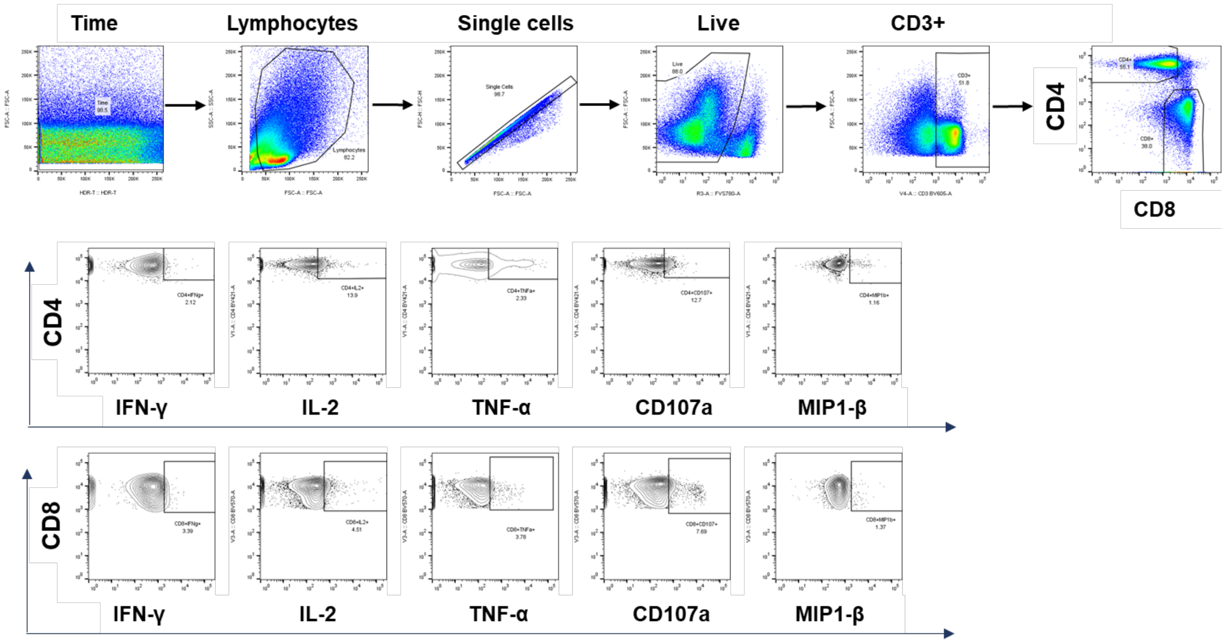

Fig S10 Gating strategy for intracellular staining flow cytometry. Lymphocytes were gated based on FSC and SSC, followed by identification of singlets and live $CD3^+$ T cells that were further gated to $CD4^+$ and $CD8^+$ T cells to determine polyfunctional expression of IFN-γ, TNF-α, IL-2, CD107a, and MIP1-β in Gag-, Env-, and Pol-peptide pool–stimulated PBMCs by Boolean gating of single marker expressing cells. Fluorescence minus one control and unstimulated samples were used to define gates for IFN-γ, TNF-α, IL-2, CD107a, and MIP1-β. Flow plots show representative responses in $CD4^+$ and $CD8^+$ T cells from one NHP.

Supplementary Table

**Cell type**	**Vaccine**	**Vaccine+Flt3L-Fc**	**Range in healthy rhesus[1]**
RBC ($\times 10^6$/µL)	WO: 5.67±0.24	WO: 5.27±0.45	3.85–7.02
	W8: 5.92±0.24	W8:5.29±0.42	
	W16:5.9±0.32	W16:5.52±0.25	
WBC ($\times 10^3$/µL)	WO:8±2.46	WO:11.2±4.70	3.1–12.1
	W8:7.5±3.05	W8:9.5±3.37	
	W16:7.6±2.78	W16:6±2.28	
Lymphocytes ($\times 10^3$/µL)	WO:3.04±1.01	WO:3.83±1.46	0.44–6.51
	W8:2.6±0.90	W8:3.38±1.25	
	W16:3.50±1.07	W16:3.43±1.37	
Neutrophils ($\times 10^3$/µL)	WO:4.48±1.94	WO:5.01±2.60	1.29–8.90
	W8:3.31±2.61	W8:4.17±2.19	
	W16:3.62±2.00	W16:1.97±1.31	
Eosinophils ($\times 10^3$/µL)	WO: 0.016±0.08	WO:0.11±0.12	0.09–0.72
	W8:0.09±0.12	W8:0.07±0.12	
	W16:0.06±0.29	W16:0.04±0.11	
Basophils ($\times 10^3$/µlL)	WO:0.01±0.003	WO:0.01±0.03	0.00–0.02
	W8:0.01±0.004	W8:0.01±0.036	
	W16:0±0.004	W16:0.01±0.005	

TABLE S1 Volumetric cell count. Cell counts of RBCs, WBCs, lymphocytes, neutrophils, eosinophils, and basophils was evaluated at week (W) 0, 8, and 16. [1]Reference ranges for healthy rhesus from Sang B-K et al., *Lab Anim Res.*, 2019.

REFERENCE

1. Koo BS, Lee DH, Kang P, Jeong KJ, Lee S, Kim K, Lee Y, Huh JW, Kim YH, Park SJ, Jin YB, Kim SU, Kim JS, Son Y, Lee SR. Reference values of hematological and biochemical parameters in young-adult cynomolgus monkey (*Macaca fascicularis*) and rhesus monkey (*Macaca mulatta*) anesthetized with ketamine hydrochloride. *Lab Anim Res* 2019;35:7.
